# Survival Outcomes Following Combination of First-Line Platinum-Based Chemotherapy with S-1 in Patients with Advanced Gastric Cancer

**DOI:** 10.3390/cancers12123780

**Published:** 2020-12-15

**Authors:** Anna Koumarianou, Anastasios Ntavatzikos, Christos Vallilas, Katerina Kampoli, Zoi Kakoseou, Michalis V. Karamouzis

**Affiliations:** 1 Hematology Oncology Unit, Fourth Department of Internal Medicine, Attikon University Hospital, Medical School, National and Kapodistrian University of Athens, 12462 Haidari, Greece; adavatzik@med.uoa.gr (A.N.); katerinakamboli@yahoo.gr (K.K.); 2 Molecular Oncology Unit, Department of Biological Chemistry & First Department of Internal Medicine, “Laiko” General Hospital, Medical School, National and Kapodistrian University of Athens, 11527 Athens, Greece; chris-vallilas@hotmail.com (C.V.); zoikak@hotmail.com (Z.K.); m_karam@otenet.gr (M.V.K.)

**Keywords:** gastric cancer, real-world data, first line, chemotherapy, oxaliplatin, tegafur, cisplatin

## Abstract

**Simple Summary:**

Regardless of recent advances in the understanding of cancer biology, gastric cancer remains one of the leading causes of death worldwide. The efficacy of tegafur/gimeracil/oteracil (S-1) combined with a platinum agent in the first-line setting in advanced gastric cancer has been previously demonstrated in randomized clinical trials. However, real-world data regarding S-1 and platinum compounds (oxaliplatin or cisplatin) in European patients remains limited. This retrospective study aimed to report on the efficacy, safety as well as dose intensity in a European population with advanced gastric cancer. Patients have received S-1 in combination with a platinum agent for six cycles of treatment. S-1 combination with oxaliplatin was associated with superior efficacy as compared to cisplatin. This study confirms that S-1 in combination with oxaliplatin in the first-line setting of advanced gastric cancer is a safe and effective treatment option in European patients.

**Abstract:**

The efficacy of S-1 combined with a platinum agent in the first-line setting and in patients with advanced gastric adenocarcinoma has been previously demonstrated in randomized clinical trials. However, real-world data regarding S-1 efficacy in European patients remains limited. In the present study, we reviewed the data of a European cohort of patients with advanced gastric cancer treated with first-line therapy consisting of S-1 in combination with a platinum agent. Forty-eight patients (29 with locally advanced/inoperable and 19 with metastatic disease) were treated with S-1 plus oxaliplatin (33 patients) or S1 plus cisplatin (15 patients). The Cox regression analysis, adjusted with propensity score, indicated that the use of cisplatin as compared to oxaliplatin was associated with increased risk of death (HR 9.634, *p* = 0.000). Four SAEs (serious adverse events) GIII were recorded (1 fatigue, 1 neutropenia, 1 anemia, 1 diarrhea) in 3 patients. S-1 combination with a platinum agent in the first-line setting in European patients with advanced gastric cancer results to similar survival outcomes and toxicity with previously reported data from Asian populations. S-1 combination with oxaliplatin seems to be associated with superior efficacy as compared to cisplatin.

## 1. Introduction

Gastric cancer (GC) was responsible for over 1,000,000 new cases in 2018 and an estimated 783,000 deaths, making it one of the most frequently diagnosed cancer and a leading cause of death in both sexes worldwide [1]. While the incidence of GC overall is declining [2], it is characterized by an increase of non-cardiac primaries and by a rise of its incidence in persons <50 years of age [3]. A recent retrospective study in the Greek population indicated that advanced clinical stage (cT4 and cN3) is associated with adverse prognosis in potentially resectable GC [4]. S-1, is a combination of three drugs, tegafur (a 5-FU prodrug), gimeracil (a dihydropyrimidine dehydrogenase inhibitor), and oteracil (an orotate phosphoribosyl transferase inhibitor) in an oral formulation designed to provide effective plasma concentrations of 5-FU. A meta-analysis comparing capecitabine to S-1 suggested that S-1 is not more effective than capecitabine in the treatment of GC patients, but does exhibit less toxicity with regard to hand-foot syndrome (HFS) [5]. Based on available evidence from phase III studies, chemotherapy including a fluoropyrimidine (5-FU, capecitabine, S-1) plus a platinum analog is considered the optimal first-line treatment in recent guidelines [6,7,8]. In the FLAGS phase III study, the combination of S-1 with cisplatin was non-inferior to fluorouracil with cisplatin in overall survival for patients with advanced GC, but was associated with significantly less HFS, hematological and gastrointestinal toxicity [9]. Comparable progression-free survival (PFS) and overall survival (OS) but a better toxicity profile for S-1 was identified in a meta-analysis of clinical trials of fluoropyrimidines including also 5-FU and capecitabine in previously untreated GC [10]. A more recent meta-analysis comparing platinum-based combination with S-1 or 5-fluorouracil indicated similar survival outcomes and response rates but longer time to treatment failure and fewer adverse events in those treated with platinum/S-1 [11]. In the pivotal phase III trial, S-1 plus oxaliplatin was shown to be as effective as S-1 plus cisplatin for advanced GC and to display fewer side effects [12].

A phase I pharmacokinetic analysis has indicated different tolerability of S-1 between Asian and Western patients due to polymorphisms of CYP2A6, responsible for metabolizing the tegafur component, supporting the use of different dosing in the two groups (40 mg/m^2^ in Asian versus 25 mg/m^2^ in Western populations) [13]. 

The existence of geographic variability of molecular substrates linked to efficacy and toxicity of oral chemotherapy underlines the need for national databases reporting on real-world data. This study aimed to report on safety and dose modification/dose intensity for patients with advanced/metastatic gastric and gastroesophageal junction adenocarcinoma who received S-1 in combination with a platinum agent for a planned six cycles of treatment.

## 2. Results

### 2.1. Patient Characteristics

Forty-eight patients with locally advanced/inoperable or metastatic GC received a median of 5.35 cycles of S-1 combined with a platinum agent in the first-line setting. Based on comorbidities or pretreatment with adjuvant therapy including cisplatin, patients were treated with S-1 in combination with either cisplatin or oxaliplatin. The clinicopathologic data of all patients and according to the concomitant platinum compound are shown in Table 1. Of all patients included in the study, 27 were considered for curative therapy but in only 5 this was achieved. The remaining number of patients were found to be inoperable during surgery due to micrometastatic disease not visualized in imaging studies. Neoadjuvant and adjuvant therapy was administered in 5 patients according to FLOT (5FU, Folinic acid, Oxaliplatin, Docetaxel) protocol [14]. Forty-eight patients received first-line therapy with a platinum agent plus S-1 until disease progression. The median number of platinum agent+ S-1 cycles delivered was 5.35 (1–6) while there was no statistically significant difference among the number of cycles according to the platinum agent (cisplatin or oxaliplatin). The median number of S-1 cycles were 5.40 (1–15) cycles when combined with oxaliplatin and 5.28 (1–24) cycles with cisplatin (a non-statistically significant difference). Of 19 patients with metastatic disease, the most common metastatic site was the liver (13 pts; 24.5%) followed by the lung (5 pts; 9.4%). Less commonly recorded metastatic sites included bones and adrenals (2 pts each 3.8%), skin, brain, and uterus (1 pt each 1.9%). Peritoneal involvement was recorded in 4 patients prior to treatment initiation (7.5%).

Analysis of significant association of patients’ characteristics with the concomitant platinum compound that was administered is shown in Table 2. Patients in whom an attempt was made to remove the disease surgically, more frequently received oxaliplatin in a non-statistically significant way (*p* = 0.058). Similarly, cisplatin correlated, non-statistically, with metastatic stage at diagnosis (*p* = 0.064) and gastric primary (*p* = 0.065). No significant associations were observed among other parameters. 

### 2.2. Side Effects

The adverse event profile of S-1 combination with a platinum agent and the need for common supportive care drugs is described in Table 3. Grade I toxicities were most commonly recorded (hematologic, gastrointestinal, and fatigue) while Grade III toxicities were observed only in 3 patients all allocated to the platinum compound.

Of the recorded supportive care drugs (EPO, g-CSF, antiemetics, and antidiarrhea), the most commonly applied drug was the need for additional antiemetics (fosaprepitant and metoclopramide). Two more findings were that the use of erythropoietin was associated significantly with cisplatin, patient’s age > 65 years and gastric primary while the use of granulocyte-colony stimulating factor (G-CSF) was associated with oxaliplatin. Contrary to platinum compounds, S-1 treatment was not associated with significant toxicities.

### 2.3. Survival Outcomes

The results of 1-year PFS/OS, 2-years PFS/OS, and the median PFS/OS of the entire cohort and according to platinum compound are reported in Table 4.

### 2.4. Cox Regression Analyses for PFS and OS

The baseline factors included in the univariable Cox regression analyses for PFS and OS included gender, age, performance status (PS) according to ECOG/WHO (0 vs. 1 and 2), stage (locally advanced vs. metastatic), number of metastasis (0/1/>1), tumor primary (esophagogastric junction vs. stomach), tumor grade (II vs. III), surgery (yes vs. no), platinum agent (cisplatin vs. oxaliplatin), supportive therapy (G-CSF and EPO), adverse events (grade 0/I/II/III, neuropathy, fatigue, anemia, diarrhea). Selected data of significance regarding the univariable analyses are shown in Table 5. Performance status 1 and 2 was adversely associated with PFS and OS as compared to PS0. Histological grade III and metastatic stage were adversely associated with PFS while oxaliplatin was positively associated with OS. More than 1 metastatic site was negatively associated with survival (PFS: HR 2.603; 95%CI: 0.735–9.214; *p* = 0.022 and OS: HR 4.849; 95%CI: 1.260–18.66; *p* = 0.022).

**Figure 1:**
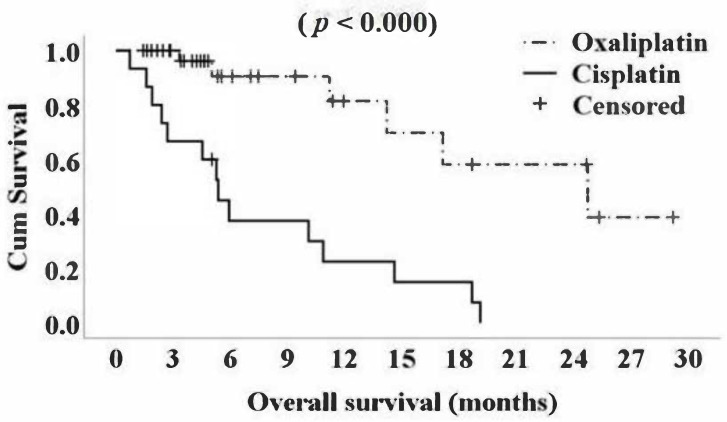
Kaplan–Meier curves for overall survival (OS) by platinum agent (Oxaliplatin vs. Cisplatin) and S-1 from treatment onset (months).

Cox regression analysis for platinum agent, adjusted to propensity score is shown in Table 6. Platinum agent was found to associate with OS. The use of oxaliplatin was found to associate positively with OS (Figure 1).

### 2.5. Other Surrogate Endpoints of Response

Disease response to treatment, ORR, and DCR are shown in Table 4. Patients with locally advanced disease were found to have significantly better disease control (RR 1.734; 95% CI 1.047–2.872; *p* = 0.024) and objective response rate (RR 2.714; 95% CI 1.073–6.865; *p* = 0.018) as compared to patients with metastatic disease.

## 3. Discussion

This study has generated real-world evidence on the impact of S-1 and a platinum agent including oxaliplatin as first-line therapy in patients with advanced GC treated in the routine care of Greece, confirming clinical efficacy and safety outcomes of the pivotal phase III study [12]. Moreover, to the best of our knowledge, this is one of the first real-world studies to provide data regarding the impact of treatment on European patients. The median PFS and OS in our study was 5.1 and 14.6 months, which is similar to that reported from previous studies [12,15,16,17]. However, the mPFS and mOS of patients receiving S-1 plus oxaliplatin was higher, compared to previously reported studies, at 8.4 and 24.7 months respectively [12,16]. On the contrary, the mPFS and mOS of patients receiving S-1 plus cisplatin was lower compared to previously reported studies at 3.7 and 5.4 months respectively [12,15]. Several patient characteristics may account for the variance in the aforementioned outcomes, while more patients have been treated with the oxaliplatin-based regimen in our cohort. The population in our study was younger and with a lower presence of liver, peritoneal, and lung metastases than those in the other studies [12,15,16]. Due to variability and imbalances in the characteristics of patients treated with cisplatin and oxaliplatin in this and other previous studies direct comparison is limited.

In our study, we did not consider histologic subtype, but we considered histologic grade and found a statistically significant difference in the PFS in the univariate analysis. In the phase III study analysis of responses to first-line chemotherapy included the histologic subtypes diffuse versus intestinal and reported similar responses [12]. Following studies have also indicated similar responses and survival outcomes between intestinal and diffuse histologic subtypes of patients treated with fluoropyrimidines and a platinum agent [18]. A population-based study including 9327 patients who survived more than 3 years has indicated that among other factors associated with longer survival included localized stage, intestinal histology surgery, regional stage, and well or moderately differentiated tumors [19].

Another important finding in our study is that patients receiving oxaliplatin and S-1 had superior survival compared to those treated with cisplatin and S-1. This is in line with a randomized open-label phase II study and a recent meta-analysis [20,21]. Contrary to our study a randomized open-label phase III study indicated equal activity of the two combinations, but this later study had a non-inferiority design [12]. SOPP, a recent phase III study of similar design also reported comparable OS in both groups but different toxicities [22]. Anemia, leucopenia, neutropenia, febrile neutropenia, and oral mucositis were more common with cisplatin and S-1 whereas thrombocytopenia, nausea, vomiting, and peripheral neuropathy were more common with oxaliplatin and S-1 [22].

In line with the survival outcome, our study indicated better objective responses in the group of patients treated with oxaliplatin and S-1 as compared to cisplatin and S-1. Similar to our study a recently published meta-analysis has indicated that oxaliplatin-based regimen is superior to a cisplatin-based regimen in tumor remission as first-line chemotherapy for advanced GC [21]. In this meta-analysis, six randomized control studies were included (3 phase III and 3 phase II) with a total of 2140 patients. Toxicity related to an oxaliplatin-based regimen was significantly superior to a cisplatin-based regimen as it could reduce the occurrence of most adverse events, but with an increased risk of thrombocytopenia, sensory neuropathy, diarrhea, fatigue, and liver dysfunction. With respect to other response endpoints, oxaliplatin-based therapy was significantly associated with higher PR rate, ORR and DCR compared to cisplatin-based therapy. Based on these findings, it was suggested that for patients with bulky disease requiring urgent symptom alleviation should be treated with the oxaliplatin-based regimen. From our retrospective study, safe conclusions on the toxicity profile of S-1 and platinum combinations cannot be made. However, we indicated neutropenia (mainly G1) in 46% of patients and an associated high supportive use of GCSF. The pivotal phase III study indicated higher rates of neutropenia 69% and 79% associated with the use of S-1 and oxaliplatin or cisplatin respectively [12]. 

When treating patients with advanced GC, special attention should be made for patients with tumors of the human epidermal growth factor receptor 2 (HER2) subtype. Patients with HER2 positive disease were not included in our study as they had not been treated with S-1 due to established anti-HER2 treatment available in this setting at the time [23]. In the pivotal TOG trial, the median OS of patients receiving trastuzumab, cisplatin and capecitabine, or 5FU was 13.8 months [23]. Later, two smaller and phase II studies including trastuzumab, cisplatin, and S-1 reported a median OS of 14.6 and 16 months respectively [24,25]. Recently, the results of a phase II trial of trastuzumab, oxaliplatin, and S-1 indicated a median OS at 18.1 months [26]. Although not directly comparable, collectively these data add in the possible superior efficacy of oxaliplatin with S-1 in advanced GC even in the HER2 positive subgroup of patients.

SOLAR, a phase III study of TAS-118 (a new oral drug containing S-1 and leucovorin) plus oxaliplatin versus S-1 plus cisplatin as first-line chemotherapy in patients with advanced GC indicated superiority in survival outcomes and less grade 3–4 neutropenia in the TAS-118 plus oxaliplatin arm over S-1 plus cisplatin [27]. The study indicated a response rate of 73% in the TAS-118 plus oxaliplatin group as compared to 50% in the S-1 plus cisplatin group underscoring the advantage of oxaliplatin combination in disease downsizing. Earlier studies have indicated that oxaliplatin, a third-generation platinum with a 1,2-diaminocyclohexane (DACH) carrier ligand, has comparable efficacy and less nephrotoxicity and gastrointestinal toxicity compared to cisplatin [28].

Overall, oxaliplatin and S-1 is a widely accepted regimen due to high efficacy, also verified in our trial, and is being used as the backbone to combine with newer agents in the 1st line setting. One such effort investigated the addition of the immune-modulatory agent pembrolizumab in the Keynote 659 phase IIb study [29]. This study was a non-randomized, multicenter, open-label phase IIb study in patients with advanced programmed death-ligand 1 positive and HER2-negative gastric and gastroesophageal junction tumors. Patients were treated with chemotherapy and pembrolizumab 200 mg until disease progression and to a maximum of 35 cycles. Although survival data are maturing, early results indicated a response rate of 74% while treatment-related adverse events were decreased platelet count (14.8%), decreased neutrophil count (13.0%), colitis (5.6%), and adrenal insufficiency (5.6%) [29]. 

Another attempt to improve oxaliplatin/S-1 results was carried out by adding the angiogenic agent ramucirumab and paclitaxel in the phase 2 RAINSTORM study [30]. The study that investigated the effect of first-line S-1 plus oxaliplatin with or without ramucirumab followed by paclitaxel plus ramucirumab on advanced GC in east Asia resulted negative for improved benefit by the addition of ramucirumab [30]. In a well-conducted phase 2, randomized, controlled trial, Hironaka and colleagues showed that S-1 plus leucovorin and oxaliplatin was better than both S-1 plus cisplatin and S-1 plus leucovorin indicating that the addition of leucovorin might provide an additional benefit when combined with S-1 plus oxaliplatin [20]. In the latter study, the comparative arm containing S-1 with leucovorin plus oxaliplatin included 47 patients of whom 66% and 34% (31 and 16 patients) achieved PR and SD respectively. In the same treatment arm, serious adverse events occurred in 18 (38%) of 47 patients; the most common grade 3–4 adverse events were neutropenia 12 (26%) of patients. Nowadays, it is well established that leucovorin (5-formyltetrahydrofolate) can increase the intracellular reduced folate pools and enhance TS enzyme inhibition by 5FU and its prodrug S-1 leading to an increase of 5FU efficacy by 5–10% [31]. In the study by Hironaka et al., the comparative arms did not contain an arm of S-1 and oxaliplatin and thus the exact benefit increase due to leucovorin cannot be calculated [20]. A study comparing S-1 with oxaliplatin±leucovorin is rather difficult to flourish as newer and more exciting studies are available in the field and the question on improved efficacy by leucovorin will remain open [32]. One important issue that may explain the differences observed in the efficacy and toxicity of chemotherapy in GC between Asian and European populations is biology. To this end, a recent genomic study revealed uncharacterized impacts of germline variants and lifestyle differences between Asian and non-Asian patients [33].

Limitations of the study are inherent due to its retrospective observational design and include potential patient-selection bias, information/recall bias, and lack of internal validity. Certain factors shown to be associated with survival outcomes, such as quality of life, in other studies have not been examined, restricting comparisons between the studies. Finally, enrollment of a small number of patients by two university hospitals from one specific region of Greece (residence to 65% of the Greek population), doesn’t align for potential variations in medical practice and generalizability of the outcomes. 

In view of the overall poor outcome of advanced GC and the low availability of resources for clinical studies, we advocate that research efforts be directed at improving the understanding of the etiopathogenesis and molecular mechanisms to develop new screening protocols and systemic treatments of this disease.

## 4. Materials and Methods

### 4.1. Ethics Approval

This retrospective study was approved by the Institutional Review Board and Ethics Committee of University General Hospital Attikon, Greece (9/16-06-2020). All participating subjects had provided informed consent for data collection and analysis from their medical files. PubMed search was carried out to assess the literature for similar studies in the field.

### 4.2. Patients

Data from medical files of patients treated from January 2010 until December 2019 were included in the study. Patients with histologically confirmed Stage III b/c, Stage IV, or relapsed esophagogastric adenocarcinoma, treated with S-1 and platinum analog in the first-line setting, were selected. Assessment of imaging response was carried out according to RECIST criteria. Data on toxicity was reported and analyzed toxicity assessment was carried out according to MEDRA. 

### 4.3. Treatment Schedules

The prescription of therapy with S-1 plus cisplatin was carried out according to the summary of product characteristics (https://www.ema.europa.eu/en/documents/product-information/teysuno-epar-product-information_en.pdf) and fell within current practice. The recommended standard dose of S-1 when administered in combination with cisplatin is 25 mg/m^2^ (expressed as tegafur content) twice daily, morning and evening, for 21 consecutive days followed by 7 days rest (1 treatment cycle). Cisplatin starting dose was 75 mg/m^2^ by intravenous infusion, administered once every 4 weeks to a maximum of 6 cycles and with the continuation of single-agent S-1 thereafter. When combined with oxaliplatin, S-1 was given the same way for the first 2 weeks of a 3-week cycle. Oxaliplatin at 100 mg/m^2^ was infused for 2 h intravenously on day 1 of each cycle, according to the treatment schedules of the phase II and the pivotal phase III trials [12,16].

### 4.4. Patient Data

Patient data were retrospectively analyzed and transferred to an anonymized database according to patient data protection regulations. Data analyses were carried out to identify toxicity and effectiveness of S-1 combined with a platinum agent in the context of 1st line setting. Analysis based on the platinum agent was included. In addition, known risk factors associated with increased toxicity as well as survival outcomes were also recorded.

### 4.5. Statistical Analyses

Association of selected clinicopathological characteristics and survival outcome (progression of disease or death) was performed using the χ2 test with a 2-sided significance of 0.05. Time-to-event distributions were estimated using the Kaplan–Meier method. For all associations, the level of statistical significance was set at a = 0.05. Overall survival (OS) was defined as the interval between initiation of 1st line chemotherapy and death of any cause. Progression-free survival (PFS) was calculated as the time from 1st line chemotherapy initiation to the date of verified progression of the disease or the date of death by any cause. Disease control rate was calculated by adding the percentages of patients achieving complete response, partial response, and stable disease. Objective response rate was calculated by the percentage of patients who achieved complete and partial response.

Surviving patients were censored at the date of last contact. The relationship of clinicopathological characteristics with OS and DFS was assessed by univariate Cox regression analysis. To account for the presence of confounding and the fact that risk factors are not equally distributed in patients who received cisplatin and those who received oxaliplatin, we estimated patients’ propensity scores. In brief, the propensity score is the patient’s probability to receive the observed treatment, given his/her characteristics, namely age, gender, PS, tumor site, stage and histological grade, and surgery. The propensity score was then included as a covariate in the Cox regression model, along with the indicator of the treatment group (oxaliplatin vs. cisplatin). All statistical analyses were performed using the SPSS software version 25.0 for Windows (SPSS Inc., Chicago, IL, USA).

## 5. Conclusions

S-1 combination with a platinum agent in the first-line setting of European patients with advanced GC results in similar survival outcomes and toxicity profile with previously reported data from Asian populations. S-1 combination with oxaliplatin is associated with superior efficacy as compared to cisplatin.

## Figures and Tables

**Table 1:** Clinicopathologic data of patients with advanced gastric cancer treated with first-line S-1 plus a platinum agent.

Clinicopathologic Data	*n* (%)	Oxaliplatin	Cisplatin	*p*
	48 (100)	33 (100)	15 (100)	
Median Age (range)	63 (24–84)	60 (24–84)	66 (47–81)	0.407
Male	37 (77.1)	25 (75.8)	12 (80.0)	1.000
Primary site				0.065
Stomach	25 (52.1)	14 (42.4)	11 (73.3)	
Gastroesophageal Junction	23 (47.9)	19 (57.6)	4 (26.7)	
Stage				0.064
Locally Advanced	29 (60.0)	23 (69.7)	6 (40.0)	
Metastatic	19 (39.6)	10 (30.3)	9 (60.0)	
Histologic grade				
II	17 (35.4)	11 (33.3)	6 (40.0)	0.749
III	31 (64.6)	22 (66.7)	9 (60.0)	
Previous Anticancer therapy				
Considered for Surgery	27 (56.3)	22 (66.7)	5 (33.3)	0.058
Perioperative Chemotherapy	5 (10.4)	5 (15.2)	0 (0.0)	0.167
PS (ECOG/WHO)				0.671
0	33 (68.8)	22 (66.7)	11 (73.3)	
1	13 (27.1)	10 (30.3)	3 (20.0)	
2	2 (4.2)	1 (3.0)	1 (6.7)	

*n*: number of patients; *p*: *p*-value; PS: performance status according to Eastern Cooperative Oncology Group/World Health Organization (ECOG/WHO).

**Table 2:** Association between patients’ characteristics and the administered chemotherapy regimen.

Patient Characteristic	Regimen with	RR (95%CI)	*p* Value
Surgery	Oxaliplatin	2.000 (0.934–4.255)	0.058
Metastatic Stage	Cisplatin	1.980 (1.021–3.846)	0.064
Primary site: Stomach	Cisplatin	1.727 (1.047–2.857)	0.065

**Table 3:** Adverse reactions and use of supportive drugs in patients with advanced or metastatic gastric cancer treated with S-1 plus a platinum agent according to platinum agent.

	N (%)	Total Grade			Oxaliplatin Grade			Cisplatin Grade		
Adverse Reaction		I	II	III	I	II	III	I	II	III
Neutropenia	22 (45.8)	18	3	1	15	0	0	3	3	1
Anemia	45 (93.7)	34	10	1	28	5	0	6	5	1
Thrombocytopenia	12 (25.0)	10	2	0	5	1	0	5	1	0
Diarrhea	29 (40.4)	27	1	1	26	0	1	1	1	0
Nausea	41 (85.4)	37	4	0	26	2	0	11	2	0
Vomiting	36 (75.0)	32	4	0	21	2	0	11	2	0
Neuropathy sensory	10 (20.8)	10	0	0	10	0	0	0	0	0
Fatigue	33 (68.7)	27	5	1	25	0	0	2	5	1
Grade III *	3 (6.3)						1 (3.0)			2 (13.3)
**Supportive drugs**										
Antiemetic	47 (97.9)						33 (100)			14 (93.3)
Antidiarrheal	3 (6.3)						3 (9.1)			0 (0.0)
G-CSF	39 (81.3)						32 (97)			7 (46.7)
Erythropoietin	20 (41.7)						8 (24.2)			12 (80)

* Number of patients with grade III toxicity; G-CSF: granulocyte colony-stimulating factor.

**Table 4:** Survival outcomes of patients with advanced gastric cancer treated with first-line combination chemotherapy and according to the platinum agent.

Survival Outcome	*n* (%)	Oxaliplatin	Cisplatin
	48 (100)	33 (100)	15 (100)
Best response			
Complete Response (CR)	6 (12.5)	6 (18.2)	0 (0.0)
Partial Response (PR)	14 (29.2)	9 (27.3)	5 (33.3)
Stable Disease (SD)	12 (25.0)	9 (27.3)	3 (20.0)
Progressive Disease (PD)	15 (31.3)	8 (24.2)	7 (46.7)
Overall survival (OS)			
Deaths	20 (41.7)	6 (18.2)	14 (93.3)
1-year OS	55.9%	81.4%	22.5%
2-year OS	26.8%	58.2%	0.0%
Median OS m (95%CI)	14.6 (8.4–20.8)	24.7 (10.1–39.3)	5.4 (3.9–6.8)
Progression - free survival (PFS)			
Events	36 (75.0)	21 (63.6)	15 (100)
1-year PFS	18.1%	18.9%	13.3%
2-year PFS	3.6%	6.3%	0.0%
Median PFS m (95%CI)	5.1 (3. 7–6.5)	8.4 (5.0–11.9)	3.7 (1.4–6.0)
Median follow up m (range)	7.1 (2.0–45.8)	5.0 (1.4–29.2)	5.3 (0.7–19.1)
ORR (CR + PR)	20 (41.7)	15 (45.5)	5 (33.3)
Median duration ORR m (95%CI)	8.2 (2.3–14.10)	8.2 (4.0–12.4)	2.9 (1.6–4.2)
DCR (CR + PR + SD)	32 (66.7)	24 (72.7)	8 (53.3)
Median duration DCR m (95%CI)	5.4 (1.1–9.7)	6.1 (5.0–7.3)	2.3 (1.6–3.0)

*n*: number of patients; CI: confidence interval; m: months ORR: objective response rate; DCR: disease control rate.

**Table 5:** Univariate Cox regression analysis for clinicopathologic features.

		PFS			OS	
Variable	HR	95%CI	*p* Value	HR	95%CI	*p* Value
PS (ECOG/WHO)						
0	1			1		
1 and 2	3.559	1.458–8.676	0.005	5.364	1.512–19.04	0.009
Fatigue Grade						
I	1			1		
II and III	6.456	2.309–18.05	0.002	113.1	12.07–1060	0.000
Sensory Neuropathy						
Grade 0	2.536	0.890–7.229	0.082	2.492	0.576–10.78	0.222
Grade 1	1			1		
Platinum Agent						
Cisplatin	1.904	0.966–3.75	0.063	6.764	2.427–18.84	0.000
Oxaliplatin	1			1		
Surgery						
No	1					
Yes	0.605	0.309–1.183	0.142	0.510	0.209–1.240	0.137
Histologic Grade						
II	1			1		
III	2.084	1.002–4.336	0.045	1.667	0.657–4.229	0.282
Stage						
Metastatic	2.049	1.032–4.065	0.040	2.099	0.868–5.079	0.100
Locally advanced	1			1		

PFS: progression-free survival; OS: overall survival; PS: performance status; HR: hazard ratio; CI: confidence interval.

**Table 6:** Cox regression survival analysis, adjusted to propensity score, for platinum agent.

		PFS			OS	
Variable	HR	95%CI	*p* Value	HR	95%CI	*p* Value
Cisplatin vs. Oxaliplatin	1			1		
Cisplatin	1.661	0.724–3.813	0.231	9.634	2.845–32.62	0.000

PFS: progression-free survival; OS: overall survival; HR: hazard ratio; CI: confidence interval.

## References

[1] Bray F., Ferlay J., Soerjomataram I., Siegel R.L., Torre L.A., Jemal A. (2018). Global cancer statistics 2018: GLOBOCAN estimates of incidence and mortality worldwide for 36 cancers in 185 countries. CA Cancer J. Clin..

[2] Balakrishnan M., George R., Sharma A., Graham D.Y. (2017). Changing Trends in Stomach Cancer throughout the World. Curr. Gastroenterol. Rep..

[3] Venerito M., Link A., Rokkas T., Malfertheiner P. (2019). Review: Gastric cancer—Clinical aspects. Helicobacter.

[4] Koumarianou A., Krivan S., Machairas N., Ntavatzikos A., Pantazis N., Schizas D., Martikos G., Kampoli K., Misiakos E.P., Patapis P. (2018). Ten-year survival outcomes of patients with potentially resectable gastric cancer: Impact of clinicopathologic and treatment-related risk factors. Ann. Gastroenterol..

[5] He A.-B., Peng X.-L., Song J., Zhang J.-X., Dong W.-G., Luo R.-F., Tang Y. (2015). Efficacy of S-1vscapecitabine for the treatment of gastric cancer: A meta-analysis. World J. Gastroenterol..

[6] Douridas G.N., Fountoulakis A., Souglakos J., Gourtsoyianni S., Vini L., Levidou G., Liakakos T., Agalianos C., Dervenis C., Kalogeridi M.A. (2020). Consensus statement of the Hellenic and Cypriot Gastric Cancer Study Group on the diagnosis, staging and management of gastric cancer. Updat. Surg..

[7] ESMO Guidelines Committee Gastric Cancer Treatment Recommendations. https://www.esmo.org/guidelines/gastrointestinal-cancers/gastric-cancer/eupdate-gastric-cancer-treatment-recommendations.

[8] National Comprehensive Cancer Network Gastric Cancer. https://www.nccn.org/professionals/physician_gls/pdf/gastric.pdf.

[9] Ajani J.A., Rodriguez W., Bodoky G., Moiseyenko V., Lichinitser M., Gorbunova V., Vynnychenko I., Garin A., Lang I., Falcon S. (2010). Multicenter Phase III Comparison of Cisplatin/S-1 With Cisplatin/Infusional Fluorouracil in Advanced Gastric or Gastroesophageal Adenocarcinoma Study: The FLAGS Trial. J. Clin. Oncol..

[10] Ter Veer E., Ngai L.L., Van Valkenhoef G., Mohammad N.H., Anderegg M.C.J., Van Oijen M.G.H., Van Laarhoven H.W.M. (2017). Capecitabine, 5-fluorouracil and S-1 based regimens for previously untreated advanced oesophagogastric cancer: A network meta-analysis. Sci. Rep..

[11] Luo D., Wang L., Chen X., Xiong Y., Yi F., Ding J., Ding H., Wei Y., Zhang W. (2020). Comparison of Platinum/S-1 and Platinum/5-Fluorouracil as First-Line Chemotherapy for Advanced Gastric or Gastroesophageal Junction Cancer: A Meta-Analysis Based on Randomized Controlled Trials. Chemotherapy.

[12] Yamada Y., Higuchi K., Nishikawa K., Gotoh M., Fuse N., Sugimoto N., Nishina T., Amagai K., Chin K., Niwa Y. (2015). Phase III study comparing oxaliplatin plus S-1 with cisplatin plus S-1 in chemotherapy-naïve patients with advanced gastric cancer. Ann. Oncol..

[13] Ajani J.A., Faust J., Ikeda K., Yao J.C., Anbe H., Carr K.L., Houghton M., Urrea P. (2005). Phase I Pharmacokinetic Study of S-1 Plus Cisplatin in Patients with Advanced Gastric Carcinoma. J. Clin. Oncol..

[14] Al-Batran S.-E., Homann N., Pauligk C., Goetze T.O., Meiler J., Kasper S., Kopp H.-G., Mayer F., Haag G.M., Luley K. (2019). Perioperative chemotherapy with fluorouracil plus leucovorin, oxaliplatin, and docetaxel versus fluorouracil or capecitabine plus cisplatin and epirubicin for locally advanced, resectable gastric or gastro-oesophageal junction adenocarcinoma (FLOT4): A randomised, phase 2/3 trial. Lancet.

[15] Koizumi W., Narahara H., Hara T., Takagane A., Akiya T., Takagi M., Miyashita K., Nishizaki T., Kobayashi O., Takiyama W. (2008). S-1 plus cisplatin versus S-1 alone for first-line treatment of advanced gastric cancer (SPIRITS trial): A phase III trial. Lancet Oncol..

[16] Koizumi W., Takiuchi H., Yamada Y., Boku N., Fuse N., Muro K., Komatsu Y., Tsuburaya A. (2009). Phase II study of oxaliplatin plus S-1 as first-line treatment for advanced gastric cancer (G-SOX study). Ann. Oncol..

[17] Koizumi W., Tanabe S., Saigenji K., Ohtsu A., Boku N., Nagashima F., Shirao K., Matsumura Y., Gotoh M. (2003). Phase I/II study of S-1 combined with cisplatin in patients with advanced gastric cancer. Br. J. Cancer.

[18] Arai H., Iwasa S., Boku N., Kawahira M., Yasui H., Masuishi T., Muro K., Minashi K., Hironaka S., Fukuda N. (2019). Fluoropyrimidine with or without platinum as first-line chemotherapy in patients with advanced gastric cancer and severe peritoneal metastasis: A multicenter retrospective study. BMC Cancer.

[19] Kunz P.L., Gubens M., Fisher G.A., Ford J.M., Lichtensztajn D.Y., Clarke C.A. (2012). Long-Term Survivors of Gastric Cancer: A California Population-Based Study. J. Clin. Oncol..

[20] Hironaka S., Sugimoto N., Yamaguchi K., Moriwaki T., Komatsu Y., Nishina T., Tsuji A., Nakajima T.E., Gotoh M., Machida N. (2016). S-1 plus leucovorin versus S-1 plus leucovorin and oxaliplatin versus S-1 plus cisplatin in patients with advanced gastric cancer: A randomised, multicentre, open-label, phase 2 trial. Lancet Oncol..

[21] Zhang F., Zhang Y., Jia Z., Wu H., Gu K. (2019). Oxaliplatin-Based Regimen is Superior to Cisplatin-Based Regimen in Tumour Remission as First-line Chemotherapy for Advanced Gastric Cancer: A Meta-Analysis. J. Cancer.

[22] Lee K.-W., Chung I.-J., Ryun P.S., Park Y.I., Nam B.-H., Oh H.-S., Lee K.H., Han H.S., Seo B.-G., Jo J.C. (2020). Multicenter phase III trial of S-1 and cisplatin versus S-1 and oxaliplatin combination chemotherapy for first-line treatment of advanced gastric cancer (SOPP trial). Gastric Cancer.

[23] Bang Y.-J., Van Cutsem E., Feyereislova A., Chung H.C., Shen L., Sawaki A., Lordick F., Ohtsu A., Omuro Y., Satoh T. (2010). Trastuzumab in combination with chemotherapy versus chemotherapy alone for treatment of HER2-positive advanced gastric or gastro-oesophageal junction cancer (ToGA): A phase 3, open-label, randomised controlled trial. Lancet.

[24] Chua C., Tan I.B., Yamada Y., Rha S.Y., Yong W.P., Ong W.S., Tham C.K., Ng M., Tai D.W.M., Iwasa S. (2015). Phase II study of trastuzumab in combination with S-1 and cisplatin in the first-line treatment of human epidermal growth factor receptor HER2-positive advanced gastric cancer. Cancer Chemother. Pharmacol..

[25] Kurokawa Y., Sugimoto N., Miwa H., Tsuda M., Nishina S., Okuda H., Imamura H., Gamoh M., Sakai D., Shimokawa T. (2014). Phase II study of trastuzumab in combination with S-1 plus cisplatin in HER2-positive gastric cancer (HERBIS-1). Br. J. Cancer.

[26] Takahari D., Chin K., Ishizuka N., Takashima A., Minashi K., Kadowaki S., Nishina T., Nakajima T.E., Amagai K., Machida N. (2019). Multicenter phase II study of trastuzumab with S-1 plus oxaliplatin for chemotherapy-naïve, HER2-positive advanced gastric cancer. Gastric Cancer.

[27] Kang Y.-K., Chin K., Chung H.C., Kadowaki S., Oh S.C., Nakayama N., Lee K.-W., Hara H., Chung I.-J., Tsuda M. (2020). S-1 plus leucovorin and oxaliplatin versus S-1 plus cisplatin as first-line therapy in patients with advanced gastric cancer (SOLAR): A randomised, open-label, phase 3 trial. Lancet Oncol..

[28] Raymond E., Chaney S.G., Taamma A., Cvitkovic E. (1998). Oxaliplatin: A review of preclinical and clinical studies. Ann. Oncol..

[29] Kawazoe A., Yamaguchi K., Yasui H., Negoro Y., Azuma M., Amagai K., Hara H., Baba H., Tsuda M., Hosaka H. (2020). Safety and efficacy of pembrolizumab in combination with S-1 plus oxaliplatin as a first-line treatment in patients with advanced gastric/gastroesophageal junction cancer: Cohort 1 data from the KEYNOTE-659 phase IIb study. Eur. J. Cancer.

[30] Yoshikawa T., Muro K., Shitara K., Oh D.-Y., Kang Y.-K., Chung H.C., Kudo T., Chin K., Kadowaki S., Hamamoto Y. (2019). Effect of First-line S-1 Plus Oxaliplatin with or without Ramucirumab Followed by Paclitaxel Plus Ramucirumab on Advanced Gastric Cancer in East Asia. JAMA Netw. Open.

[31] Youcef M.R. (2004). Thymidylate synthase: A critical target in cancer therapy?. Front. Biosci..

[32] Ter Veer E., Van Oijen M.G.H., Van Laarhoven H.W.M. (2016). S-1 with leucovorin and oxaliplatin for advanced gastric cancer. Lancet Oncol..

[33] Suzuki A., Katoh H., Komura D., Kakiuchi M., Tagashira A., Yamamoto S., Tatsuno K., Ueda H., Nagae G., Fukuda S. (2020). Defined lifestyle and germline factors predispose Asian populations to gastric cancer. Sci. Adv..

